# The Rubber Dam

**Published:** 1874-11

**Authors:** 


### ARTICLE IV.

### *The Rubber Dam.*

Many and great are the difficulties which dentists have been compelled to meet and contend with, in the prosecution of their arduous duties, through all the past history of the profession.

Many of these difficulties are intrinsic to the operations themselves, and are quite enough to tax all the skill, patience, and nervous force the practitioner possesses.

But in addition to these are others intimately connected with, though not part of the operations, which materially increase the labor, and in large measure detract from the perfection of the result, in consquence of the limited control which it has been possible to gain over them.

One among these, and perhaps the greatest, is the danger of inundation from saliva, and the consequent ruin of the work. Hours of valuable time have been spent and lost in unintermitted battle with this aggressive foe. If the conflict resulted in victory, that victory was shorn of much of its pleasure by the excessive weariness consequent upon the protracted struggle which preceded it. If in defeat, the mortification of that defeat was intensified by the exhaustive waste of nervous force, and the terrible prostration which was its natural result.

Numerous methods and appliances have been devised and introduced, through the years past, for the purpose of controlling this difficulty. By the faithful and persistent use of some of them, a degree of success has been achieved, and good operations performed, though always at the expense of great labor to the practitioners, and great weariness to the patient. Oftentimes tedious operations, such as are termed "building up," or making "contour fillings," have been brought nearly to completion, when in a moment, by some slight movement of the patient, or the sudden saturation of a napkin, or a copious discharge from a neighboring gland, a "tidal wave" has swept over the structure, and in a twinkling the whole been ruined.

Clamps for compressing the duct of Steno; tourniquets for holding coils of linen, or compress of various materials; pumps for removing the accumulated saliva; wooden wedges driven tightly between the teeth to dam up the watery emissions; waxed ligatures wound closely around the necks of teeth; tongue holders; indeed, almost every conceivable contrivance has been tried, with varying degrees of success, but still the obstacle remained, a sore vexation and hindrance to the dentist whose aim was thoroughness and durability.

But at length a bright day dawned—a day of conquest, of rejoicing, of relief. A happy thought entered the mind of Dr. S. C. Barnum, of New York City. Acting upon it, he devised and introduced a system of dealing with the dentist's arch enemy—saliva—at once practical and certain. This method consisted of using a thin sheet of India rubber, a material absolutely impervious to moisture, and so enveloping the tooth to be operated on, that perfect dryness can be secured during the longest operation.

After testing his new discovery, and proving its efficacy, he most generously gave it to the profession without restriction and without charge—a noble, unselfish act. Since that day blessings innumerable have been invoked upon his head by dentists and patients. Increased facility in the use of the Rubber Dam has marked the passing months, until it is daily demonstrated that the appliance is capable of universal application and worthy of universal trust. Neither is it too much to assert, perhaps, that no new thing has been introduced to the profession, among all the vast array of inventions for ten years, which has been productive of so much comfort, and such good results, as Dr. Barnum's Rubber Dam.

This being the case, it may not be amiss to consider its virtues somewhat critically.

*By the use of the Rubber Dam greater thoroughness of operations is secured.*

1st. In the preparation of cavities.

No cavity can be perfectly excavated and prepared, unless every part of it is clearly seen. When moisture continually fills the cavity, only an imperfect view of it can be obtained.

Rays of light, as is well known, are refracted by passing through fluids, so that, in looking into a vessel filled with water, the sight does not strike directly upon the point at which it is aimed, but upon another; for the line of vision is refracted or bent when it strikes the surface of the water, and the beholder is deceived; but when the vessel is empty the line of vision is direct from the eye to the precise point looked at. Now, if a cavity is filled with saliva, it will be at once perceived that there is less certainty of every point in that cavity being distinctly seen, and if not distinctly seen, then of course not surely reached by the preparing instrument.

Again, if the floor or walls of the cavity are covered with moisture, although not to such a degree as to refract the rays of visual light, still a glistening surface is given to the interior, which in a measure conceals or obscures it, and its exact condition is not so perfectly determined as when absolutely dry. This will not be disputed.

Now in preparing a cavity located on an approximal surface of a tooth, there is a constant invasion of saliva, producing the unfavorable results already referred to, and necessitating the continuous use of some absorbent, such as spunk or paper, to obviate the difficulty. But let the Rubber Dam be applied, and no hindrance from encroaching moisture is encountered. The entire interior of the cavity is clearly seen, its condition certainly determined, the instrument applied at the very point where it is needed, and a satisfactory preparation secured in all cases.

It is also contended that the application of the Rubber Dam to teeth, previous to preparation, reduces the pain of excavating sensitive dentine. Whether this is in consequence of the cut ends of the tubuli emptying themselves into the dry cavity, thus reducing the quantity of nervous fluid (if it be a fluid) which they contain, and consequently losing something of their conducting power, or whether the acuteness of sensibility which the said nervous fluid (?) possesses is somewhat obtuned by the unintermitted contact of atmospheric air, or whatever may be the cause, the desirable result is the immediate product of the Rubber Dam, and is to be set down to its credit.

When a cavity is prepared, and the filling ready to be introduced, no moisture can creep in under the gold· every piece can be placed just where it is desired; it can be thoroughly compacted; perfect cohesion of all the layers can be uniformly gained from first to last; no undue haste need interfere with the bestowal of all the care which is required, no premature termination of the operation is rendered necessary by the approach of the insidious foe, but the last piece can be put in as deliberately as the first, and the whole finished to the mind of the operator.

There is an array of advantages sufficient to commend any new thing, and it is no idle boast to claim all these for the Rubber Dam. But we can go a step further and consider the next of the trinity of desirable ends.

2d. By the use of the Rubber Dam the comfort of patients is greatly promoted.

No packing of the mouth with napkins is required. These distress a patient exceedingly, and are often quite painful, as well as uncomfortable.

No clamps upon the tongue or cheeks are necessary. The sense of being held in a vise is not experienced.

No cramping and straining of the muscles of the cheek is felt.

The terrible weariness caused by long continued holding of the mouth open, with no possibility of closing it through a tedious operation, is spared the patient, for he can occasionally close it without damaging the operation, and thus obtain most grateful relief.

The fear of strangling by reason of labored and imperfect deglutition is avoided, for the patient can swallow at frequent intervals, and in a natural manner.

The fear of spoiling the operation by an injudicious or necessary motion is not felt by the patient. He can be assured that no danger can result, so that his mind remains undisturbed through the entire sitting. And this is no slight matter. Patients often express themselves as completely "wearied out" by the constant apprehension that they might in some incautious manner do fatal damage to the operation. Take away from them this anxiety, and much is done for their comfort while they are in the chair.

This long army of good results, be it remembered, is to be set down to the credit of the Rubber Dam.

Next, expedition is promoted by the use of this appliance.

No time is lost in constructing and operating defences against saliva. The whole attention of the dentist can be devoted to his work. Having a feeling of security, his manipulations are without nervous haste, well directed and effectual, and the result is more rapid progress toward completion. Again, the operator's mind being free from apprehension, there is no diversion of his thoughts. He places his gold just where he desires, and drives it home, while the satisfaction he experiences at the perfect control he has over the case imparts celerity to his hand, and the result is expedition.

Experience in the use of this appliance will enable the dentist to adjust it in every case, and the new inventions of clamps, together with the method, already familiar, of using ligatures about the enveloped teeth, will effectually secure it in place, and present to the eye and hand of the operator, the objective point of his labor.

Is it not necessary at this day to enter into details as to the *modus operandi* of applying the Dam. The profession pretty generally understand these details. It may not be amiss, however, to give a few suggestions drawn from experience.

A large sheet is more convenient than a small one. The ends hang out over the shoulders, and the curtain falls down over the chin so far as to retain their place and keep out of the way. Small pieces are continually curling up and obstructing the sight, or presenting an advantage to the saliva, unless the piece is constantly held.

Several teeth besides the one or more to be operated on should be enclosed by the rubber, as a better view is obtained, and less hindrance experienced by folds of the appliance. On short or conical crowns, where the shape is unfavorable for holding the Dam, one of the recent improved clamps, slipped down to the neck of the tooth over the rubber, will hold it effectually.

The ingenuity of the dentist will suggest various little devices for the conquest of minor difficulties which may arise in connection with the use of the Dam, by the employment of which they will vanish before him. And to the ingenuity of the dentist these must be left.

To sum up it may be said: By a progressive and faithful dentist, nothing valuable is disregarded, and among all valuable adjuncts and accesssories placed at his disposal, he will find none more promotive of good to himself and his patients than Dr. Barnum's Rubber Dam.—*Dental Miscellany.*

